# Visceral leishmaniasis and COVID-19 coinfection – A case report

**DOI:** 10.1016/j.idcr.2021.e01358

**Published:** 2021-12-06

**Authors:** Antonis Pikoulas, Evangelia-Theophano Piperaki, Gregory Spanakos, Anastasios Kallianos, Despoina Mparmparousi, Gianna Rentziou, Georgia Trakada

**Affiliations:** a Department of Clinical Therapeutics, National and Kapodistrian University of Athens, School of Medicine, Athens, Greece; b Department of Medicine, University of Crete and Foundation for Research and Technology, Heraklion, Greece; c Department of Microbiology, Medical School, National and Kapodistrian University of Athens, School of Medicine, Athens, Greece; d National Public Health Organization, Vari, Greece

**Keywords:** Visceral leishmaniasis, SARS-CoV2, COVID-19, Co-infection

## Abstract

As the COVID-19 pandemic spreads across the globe, it will undoubtedly cross paths with long endemic infectious diseases in different areas. Interactions between SARS-CoV2 and well-known pathogens will likely give rise to unfamiliar clinical presentations, depending on complex and as yet unknown immunological interactions. We present a case of coinfection with COVI19 and visceral leishmaniasis and discuss recent reports regarding coexistence of SARS-CoV2 and *Leishmania* spp. to date.

## Introduction

In late 2019, a novel coronavirus designated as severe acute respiratory syndrome coronavirus 2 (SARS-CoV2) emerged in China. Highly transmittable via respiratory droplets and direct contact, the new virus soon achieved pandemic status [1]. As of the start of October 2021, WHO estimates there have been approximately 235,5 million confirmed cases and more than 4,8 million deaths (https://covid19.who.int/). Sweeping across the globe, the emerging virus inevitably enters territories where other infectious diseases have long been endemic and its interactions with them will undoubtedly give rise to unfamiliar clinical presentations.

Here, we present a case of coinfection with COVID-19 and visceral leishmaniasis in a patient from the Middle East, an area of high endemicity for leishmaniasis.

The leishmaniases are a group of vector-borne diseases, endemic in tropical and subtropical areas, caused by 20 different species of the intracellular parasitic genus *Leishmania*. They occur in three main clinical forms: cutaneous (CL) mucocutaneous (MCL) and visceral leishmaniasis (VL). CL is the most prevalent form, with an estimated annual incidence of 600,000–1 million cases whereas VL the most severe, life threatening form of the disease with 50,000–90,000 estimated cases annually (https://www.who.int/news-room/fact-sheets/detail/leishmaniasis).

## Case presentation

A 22-year-old woman presented to the emergency room (ER) of a tertiary hospital in Athens, in May 2021, due to diarrhea for the last 5 days and fever up to 38 °C on the last day. The patient had no immunodeficiency nor reported previous illness or comorbidities. She was an immigrant from Afghanistan, living in Athens Greece for the past 3 years. A PCR test for COVID-19 in a nasopharyngeal swab sample was performed and the patient was found positive for SARS-CoV2. Physical examination revealed tender peripheral lymphadenopathy (cervical, axillary and inguinal) and moderate hepatosplenomegaly. Vital signs were: heart rate 68 bpm, respiratory rate 22/min, blood pressure 130/72 mmHg, oxygen saturation 97% and a body temperature of 37.4 °C. Chest X-Ray was normal with no findings of pneumonia.

A complete blood count revealed pancytopenia [Hgb 9.3 g/dL, RBCs 3.53 × 106/μL, WBC 2.2 × 103/μL (Neutrophils 1.1 × 103/μL, Lymphocytes 0.9 × 103/μL, and Monocytes 0.06 × 103/μL), Platelets 102 × 103/μL]. Liver function tests were abnormal AST:165 U/L, ALT:84 U/L, INR: 1,71 and Albumin: 3.5 g/dL. Giant platelets and monocytosis were observed in the peripheral blood smear.

The patient was admitted for investigation and management, with a differential diagnosis that included hematological malignancies, bacterial and viral infections and leishmaniasis. CT scan confirmed hepatosplenomegaly (spleen 15.7 cm and liver 17.5 cm) and ruled out abdominal lymphadenopathy. During hospitalization, pancytopenia deteriorated (Hgb 7.9 g/dL, RBCs 2.98 × 106/μL, WBCs 2 × 103/μL, Platelets 77 × 103/μL). PCR assays in blood samples for HIV, HBV, HCV, CMV, EBV, HSV 1&2 and Parvovirus B19 virus were all negative. Skin Tuberculin test (PPD) and Rose Bengal agglutination test were also negative. Serology was negative for Leptospira spp., Coxiella spp., Rickettsia spp., and Plasmodium spp and positive for Leishmania (anti-Leishmania antibodies were detected in the patient’s serum using ELISA method).

**Fig. 1:**
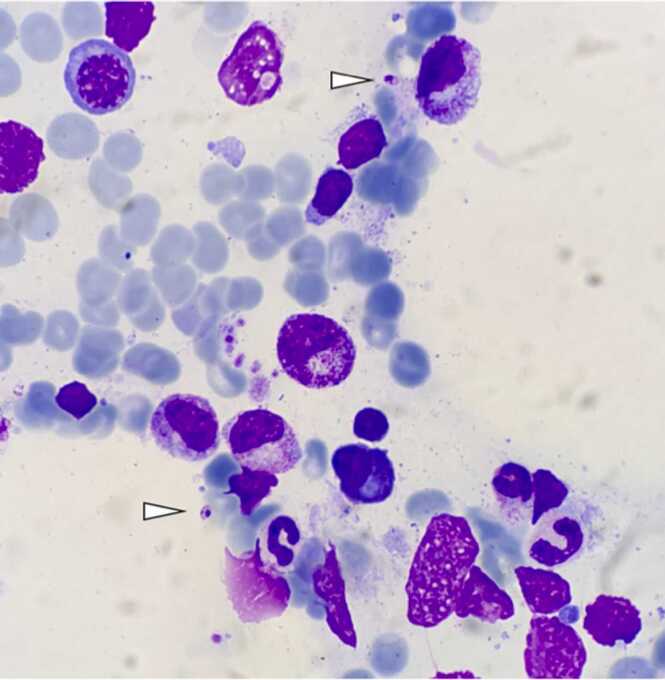
Bone marrow smear. Leishmania amastigotes can be seen (white arrows). Giemsa staining, 1000× magnification.

A bone marrow biopsy was performed. Giemsa stained smears revealed amastigotes of Leishmania [Fig. 1], and real-time PCR assay in both blood and bone marrow detected Leishmania spp [2]. Genus-specific PCR, followed by RFLP identified the parasite as L. infantum [3]. Sequencing of the PCR product confirmed that the sequence belonged to L. infantum. With the diagnosis of VL confirmed, the patient was treated with liposomal amphotericin B (daily dose of 3 mg/kg on days 1–5, day 14 and day 21, to a total dose of 21 mg/kg).

Regarding the SARS-CoV2 infection, the patient was treated with dexamethasone, enoxaparin and two doses of Remdesivir (200 mg and 100 mg, consequently discontinued due to an increase in AST and ALT). The patient remained in good clinical condition, without need for supplemental oxygen and was discharged 13 days after her admission.

## Discussion

Visceral leishmaniasis remains a major health problem in many areas of the world, usually presenting with fever, weight loss, weakness, and frequently pancytopenia [4]. COVID-19, on the other hand, is an emerging infection with clinical manifestations varying from a mild cold, to life-threatening, rapidly progressing systemic disease, that may lead to multi-organ failure [5]. During the current pandemic, the paths of these two infections will inescapably cross, with as yet unknown consequences. The protective, antiviral immune response against SARS-CoV2 is based on IFN-γ production and the resulting NK and CD8+ T cell activation, as is the protective Th1 immune response against *Leishmania*. Although it seems reasonable that immune activation against one of these infections could also protect from the other, the interaction of immune responses is far more complex and the end result remains uncertain.

A recent publication that investigated the incidence, morbidity and mortality burden of COVID-19 in patients with and without previous cutaneous leishmaniasis (CL), has found that those with cured CL had significant cross-protection from COVID-19 [6]. Th1 cellular response, mediated by IFN-γ production and leading to macrophage activation and NO production, plays a critical role in the protective immunity against *Leishmania*. This is the immunological profile usually observed in self – healing cutaneous leishmaniasis. On the other hand, a dominance of Th2 and Treg responses, associated with high IL-4, IL-5, TGF-β, and IL-10 production and a strong humoral response, is related to leishmaniasis progression as observed in chronic, progressive visceral leishmaniasis (VL) [7].

Coinfection with VL and COVID-19 has been reported once previously, in a 79-year-old male immunocompromised patient, with multiple comorbidities [8] in whom the outcome was fatal, due to respiratory failure, brought on by SARS-CoV-2. In our case, the patient was an immunocompetent female in her twenties, in whom COVID-19 followed a mild course without any complications. Regarding a possible impact that VL may have had on the severity of COVID-19 and vice versa, given the completely different epidemiological background of the two cases, and the scarceness of other reported cases to date, we can only postulate, until more data are available.

Taking into account the commonalities and differences between the protective response against SARS-CoV-2 and *Leishmania,* while also keeping in mind that immune interactions are far more complex and remain mostly undeciphered, a reasonable hypothesis in our case is that COVID-19 may have led to re-activation of a hitherto asymptomatic leishmaniasis. Re-polarization towards Th1 to face the virus may have led to parasite escape of the immune surveillance, leading to symptomatic VL. There are many cases reporting that COVID-19 has led to re-activation of chronic, asymptomatic infections caused by viruses like VZV, EBV, CMV, HSV, HHV6, HBV [9], [10], [11], protozoa [12] and fungi [13]. On the other hand, VL may have led to a specific polarization of the immune response, that rendered the patient more susceptible to viral infections, including the widely circulating COVID-19, which was transmitted to the patient during the pandemic. Currently available data do not allow us to decide which scenario might be correct. Whichever the case, there are clear indications that the two infections likely lead to complex immunological interactions, when their paths cross. In the case of CL, there is preliminary data that this interaction may be beneficial in terms of protection from COVID-19 incidence and severity. Further studies are needed to explore the association between other forms of leishmaniasis, particularly VL and COVID-19, to help in the planning of future treatment and control strategies.

## Author agreement

All authors have seen and approved the manuscript.

## Author statement

All authors have participated equal and had no conflict of interest.
